# Utilisation and impact of predict prostate on decision‐making among clinicians and patients in a specialist tertiary referral centre: A retrospective cohort study

**DOI:** 10.1002/bco2.311

**Published:** 2023-11-20

**Authors:** Madhumitha Pandiaraja, Isolde Pryle, Leah West, Lucy Gardner, Olivia Shallcross, June Tay, Nimish Shah, Vincent Gnanapragasam, Benjamin W. Lamb

**Affiliations:** ^1^ School of Clinical Medicine University of Cambridge Cambridge UK; ^2^ Department of Urology Cambridge University Hospitals NHS Foundation Trust Cambridge UK; ^3^ Academic Urology Group University of Cambridge Cambridge UK; ^4^ Department of Urology Barts Health NHS Trust London UK; ^5^ Department of Urology University College London Hospitals NHS Foundation Trust London UK; ^6^ Barts Cancer Institute Queen Mary University London London UK

**Keywords:** decision regret, patient counselling, prostate cancer, risk communication, shared decision‐making

## Abstract

**Background:**

Patients with intermediate‐risk prostate cancer are faced with the decision of whether to undergo radical treatment. Decision‐making aids, such as Predict Prostate, can empower both clinicians and patients to make treatment decisions with personalised information, but their impact on multi‐disciplinary team (MDT) decision‐making and uptake of radical treatment remains unknown.

**Objective:**

The objective of this study is to assess the utilisation and utility of Predict Prostate in informing treatment decisions for patients with intermediate‐risk prostate cancer.

**Patients and Methods:**

A retrospective cohort study was conducted in Cambridge University Hospitals (CUH) of patients referred to the prostate cancer specialist multi‐disciplinary team (pcSMDT) and robotic prostatectomy clinic (ROPD) between September 2019 and August 2021 for consideration of radical prostatectomy (RARP). Data on patient characteristics, use of PredictProstate and management decisions were collected from the Epic electronic medical record (EMR) of 839 patients, of whom 386 had intermediate‐risk prostate cancer.

**Results:**

The use of Predict Prostate at the pcSMDT increased in the second half of the study period (34.5% vs. 23.8%, *p* < 0.001). The use of Predict Prostate was associated with an increased likelihood of attending ROPD for men with CPG2 prostate cancer (OR = 2.155, 95% CI = 1.158–4.013, *p* = 0.015) but a reduced likelihood of proceeding with RARP for men with CPG2 (OR = 0.397, 95% CI = 0.209–0.753, *p* = 0.005) and CPG3 (OR = 0.305, 95% CI = 0.108–0.861, *p* = 0.025) prostate cancer.

**Conclusion:**

Our study showed that the use of Predict Prostate for patients with intermediate‐risk prostate cancer is associated with increased attendance at specialist surgical clinic and a reduced chance of undergoing radical prostate surgery.

## 1 INTRODUCTION

Prostate cancer is the most common cancer in men in the United Kingdom with incidence predicted to rise.^1^ The mortality risk from prostate cancer varies greatly depending on disease stage, from 100% 5‐year survival for Stage 1 disease to 49% survival for Stage 4 disease.^2^ Patients with intermediate‐risk, localised prostate cancer must decide between radical treatment, which includes prostatectomy and radiotherapy, and expectant management—referred to as ‘active surveillance’.

In choosing between active surveillance and radical treatment, patients are faced with the difficult task of balancing the mortality benefit offered by treatment against the risks of sexual dysfunction and urinary incontinence that accompanies surgery or radiation therapy.^3^, ^4^ Opting for active surveillance, however, can cause uncertainty about the risk of disease progression.^5^

Current recommendations for the management of primary non‐metastatic prostate cancer in the United Kingdom are based on the five‐tier Cambridge Prognostic Group (CPG) risk model, superseding the traditional three‐tiered classification system of low, intermediate and high risk. Under the CPG model, patients previously classified as ‘intermediate risk’ fall largely within CPG2 and CPG3. The National Institute for Health and Care Excellence (NICE) guidance recommends offering active surveillance as an equivalent option alongside radical treatment for CPG2 patients and as an alternative option for CPG3 patients who do not want immediate active treatment. NICE recommends that clinicians use decision tools to counsel patients with lower risk prostate cancer about management options.^6^

Predict Prostate is an online prognostication tool that uses patient characteristics to give personalised prostate cancer‐specific and overall mortality outcomes following either radical treatment or conservative management.^7^ The use of Predict Prostate has been demonstrated to lessen patient decisional conflict and provide patients with a more realistic perception of their prognosis, but its impact on final treatment decisions has not yet been evaluated.^8^ It is hypothesised that such tools may encourage these patients to avoid or defer radical treatment.

Since 2020, Predict Prostate was introduced routinely into the prostate cancer specialist multidisciplinary team (pcSMDT) meeting at Cambridge University Hospitals (CUH) to inform management recommendations. Documentation from the pcSMDT, including the output of Predict Prostate, is then used to counsel men attending the robotic prostatectomy clinic (ROPD) during discussions about management, including whether to proceed with robot‐assisted radical prostatectomy (RARP). The aim of this study was to evaluate the effect of Predict Prostate on clinical decision‐making and final treatment decisions. Specifically, the objectives were as follows:

1. To assess the uptake of Predict Prostate in the pcSMDT meeting.
2. To assess whether there was an association between utilisation of Predict Prostate and invitation to ROPD for surgical counselling.
3. To assess whether there was an association between utilisation of Predict Prostate and uptake of RARP.

## 2 PATIENTS AND METHODS

### 2.1 Study design and setting

This was a retrospective, single‐centre cohort study of patients with intermediate risk, localised, previously untreated prostate cancer who were referred to the pcSMDT service at CUH, for consideration of RARP, between September 2019 and August 2021. This study period was chosen to evaluate how the utilisation of Predict Prostate in the pcSMDT has evolved since the resource was endorsed by NICE in late 2019 and formally introduced at CUH in 2020. Patients who had been referred to the pcSMDT without cancer or with incomplete cancer staging information were excluded from the study.

The period from September 2019 to August 2020 prior to the introduction of Predict Prostate was classified as the pre‐introduction cohort and the period from September 2020 to August 2021 as the post‐introduction cohort. As the tool was intended to assist men deciding between active surveillance and radical treatment, each patient was assigned to the appropriate CPG using serum prostate specific antigen (PSA) level, clinical T stage and histological Grade Group. Only patients falling into CPG2 and CPG3 were included in comparative analyses.

The study was registered with the CUH Clinical Audit Department. Patient consent was not required due to the retrospective nature of the study. No formal ethics approval was sought as the study only involved evaluation of routine, anonymised patient data.

### 2.2 Data

Routine, prospectively recorded demographic and clinical data were retrospectively extracted from patients' electronic medical records on Epic (Epic Systems Corporation, Verona, WI). Data collected included source of pcSMDT referral, pcSMDT meeting date, patient age, serum PSA, clinical TNM stage, histological Grade Group and use of Predict Prostate at the pcSMDT meeting. In addition, information pertaining to the patients' further clinical encounters was gathered, including date of outpatient surgical clinic appointment, if any, and date of RARP, if any. If either the clinic appointment or surgery was cancelled, this was also recorded.

### 2.3 Statistical analysis

Descriptive statistics were expressed as means with standard deviations for normally distributed data and as medians with interquartile ranges for skewed distributions. For categorical variables and continuous variables, the chi‐squared test was applied to assess differences between groups. Multivariate analysis was performed using the binary logistic regression model. All statistical analyses were performed using SPSS Statistics, Version 28 (IBM Corporation, Armonk, NY, United States) and Microsoft Excel.

## 3 RESULTS

### 3.1 Patient characteristics

A total of 839 patients were included for data extraction. Five of 839 patients were excluded as they had incomplete clinical data and thus could not be assigned a CPG category, leaving a final cohort of 834 patients for analysis. Overall, the patient cohort consisted of 143 CPG1 patients (17%), 265 CPG2 patients (32%), 121 CPG3 patients (15%), 221 CPG4 patients (26%) and 84 CPG5 patients (10%). Demographic data and tumour characteristics of the patient cohort are presented in Table 1.

**TABLE 1:** Demographic data and tumour characteristics of study cohort by CPG.

	CPG1 (*n* = 143)	CPG 2 (*n* = 265)	CPG 3 (*n* = 121)	CPG 4 (*n* = 221)	CPG 5 (*n* = 84)
Age group (years), *n* (%)					
<60	50 (35.0)	60 (22.6)	18 (14.9)	35 (15.8)	14 (16.7)
60–69	68 (47.6)	123 (46.4)	67 (55.4)	113 (51.1)	37 (44.0)
70–79	25 (17.5)	81 (30.6)	35 (28.9)	70 (31.7)	29 (34.5)
≥80	‐	1 (0.4)	1 (0.8)	3 (1.4)	4 (4.8)
Median PSA (ng/ml)	5.86	7.12	10.7	8.09	15
Clinical T stage, *n* (%)					
T1	6 (4.2)	11 (4.2)	1 (0.8)	2 (0.9)	‐
T2	137 (95.8)	246 (92.8)	120 (99.2)	35 (15.8)	9 (10.7)
T3	‐	1 (0.4)	‐	184 (83.3)	71 (84.5)
T4	‐	‐	‐	‐	3 (3.6)
Tx	‐	7 (2.6)	‐	‐	1 (1.2)
GGG, *n* (%)					
1	143 (100.0)	45 (17.0)	‐	35 (15.8)	4 (4.8)
2	‐	219 (82.6)	55 (45.5)	123 (55.7)	7 (8.3)
3	‐	‐	66 (54.5)	43 (19.5)	8 (9.5)
4	‐	‐	‐	13 (5.9)	33 (39.3)
5	‐	‐	‐	‐	28 (33.3)

Abbreviations: CPG, Cambridge Prognostic Group; GGG, Gleason Grade Group; PSA, prostate specific antigen.

### 3.2 Utilisation of predict prostate

**FIGURE 1:**
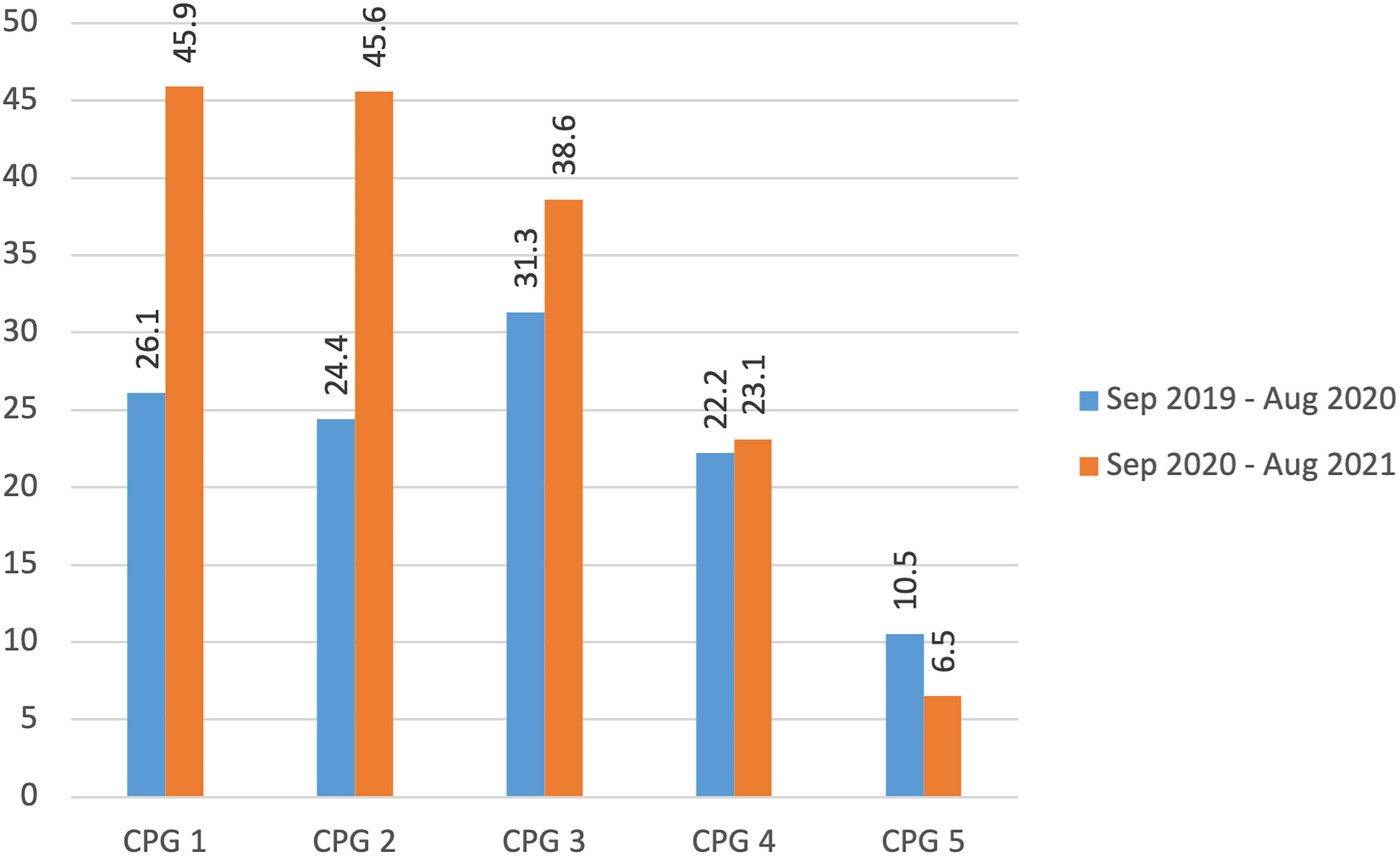
Proportion of patients in each Cambridge Prognostic Group assessed using Predict Prostate before and after formal introduction of the tool at CUH.

The proportion of patients with documentation of Predict Prostate use at the pcSMDT saw a significant increase from 23.8% to 34.7% (*p* < 0.001) between the pre‐introduction and post‐introduction cohort. This difference was greatest for CPG1 and CPG2 patients, who saw an increase in utilisation of the Predict Prostate tool of 19.9% and 21.2%, respectively (Figure 1).

### 3.3 Impact of Predict Prostate on referral to surgical outpatient clinic for patients with CPG2 and CPG3 disease

There was a significant increase in the rate of surgical clinic referral for CPG2 patients when Predict Prostate was used in the pcSMDT meeting (62.6% vs. 79.6%; *p* = 0.004) but not among CPG3 patients (65.8% vs. 81%; *p* = 0.081) (Table 2).

**TABLE 2:** Comparison of frequency of attendance at robotic prostatectomy clinic (ROPD) (upper panel) and robot‐assisted prostatectomy (RARP) (lower panel) based on usage of PredictProstate at pcSMDT stratified by Cambridge Prognostic Group (CPG).

Risk group	ROPD	PredictProstate not used, *n* (%)	PredictProstate used, *n* (%)	$\chi^{2}$	df	*p* value
CPG 2	No	64 (37.4)	19 (20.4)	8.073	1	0.004*
	Yes	107 (62.6)	74 (79.6)			
CPG 3	No	27 (34.2)	8 (19.0)	3.053	1	0.081
	Yes	52 (65.8)	34 (81.0)			

Risk group	RARP	PredictProstate not used, *n* (%)	PredictProstate used, *n* (%)	$\chi^{2}$	df	*p* value
CPG 2	No	49 (45.8)	49 (66.2)	7.348	1	0.007*
	Yes	58 (54.2)	25 (33.8)			
CPG 3	No	13 (25.0)	15 (44.1)	3.422	1	0.064
	Yes	39 (75.0)	19 (55.9)			

* Statistically significant at *p* < 0.05 level.

### 3.4 Impact of Predict Prostate on treatment decisions

The uptake of RARP among CPG2 patients was significantly lower when Predict Prostate was used at their case discussion at the pcSMDT (33.8% vs. 54.2%; *p* = 0.007). This was despite the higher frequency of surgical outpatient clinic appointments with Predict Prostate usage. There was no significant difference for patients with CPG3 in uptake of RARP depending on whether Predict Prostate was used (55.9% vs. 75%; p = 0.064) (Table 2).

### 3.5 Interrogation of drivers of decision making

Table 3 displays results of binary logistic regression analysis for predictors of attendance at surgical clinic and decision to undergo RARP for patients with CPG2 and CPG3 prostate cancer discussed at the pcSMDT. For patients with CPG2 prostate cancer, a higher likelihood of attending surgical clinic was predicted by a younger age, higher T‐stage, and using Predict Prostate; undergoing RARP was predicted by a younger age and higher grade group; use of Predict Prostate predicted a reduced chance of undertaking RARP. For patients with CPG3 prostate cancer, a higher likelihood of attending surgical clinic was predicted by a younger age only; a higher likelihood of undergoing RARP was predicted by a younger age, whereas use of Predict Prostate predicted a reduced chance of undertaking RARP.

**TABLE 3:** Results of binary logistic regression analysis for predictors of attendance at surgical clinic and decision to undergo RARP for men with CPG 2 and CPG 3 localised prostate cancer discussed at pcSMDT.

Variables	Surgical clinic attendance		Decision to undertake RARP	
	Odds ratio (95% CI)	*p* value	Odds ratio (95% CI)	*p* value
CPG 2				
Age	0.917 (0.874–0.962)	<0.001*	0.944 (0.899–0.991)	0.021*
PSA	0.976 (0.850–1.119)	0.725	1.103 (0.947–1.284)	0.208
GGG	0.775 (0.232–2.584)	0.678	4.304 (1.013–18.293)	0.048*
T‐stage	5.384 (1.278–22.692)	0.022*	–	–
PredPros	2.155 (1.158–4.013)	0.015*	0.397 (0.209–0.753)	0.005*
CPG 3				
Age	0.865 (0.797–0.938)	<0.001*	0.859 (0.775–0.951)	0.003*
PSA	1.057 (0.930–1.203)	0.395	1.088 (0.932–1.269)	0.286
GGG	1.339 (0.460–3.902)	0.592	2.956 (0.852–10.256)	0.088
T‐stage	–	–	–	–
PredPros	1.932 (0.746–5.005)	0.175	0.305 (0.108–0.861)	0.025*

Abbreviations: CPG, Cambridge Prognostic Group; GGG, Gleason Grade Group; PredPros, Predict Prostate; PSA, prostate specific antigen; RARP, robot‐assisted radical prostatectomy.

* Statistically significant at *p* < 0.05 level.

## 4 DISCUSSION

The aim of the study was to evaluate the frequency of usage of the Predict Prostate tool at CUH following NICE endorsement and formal introduction to the pcSMDT service and to understand how this affected the management pathway of patients with intermediate‐risk prostate cancer. The study demonstrated that Predict Prostate can be feasibly applied in MDT discussions and was used more frequently following its integration into routine clinical practice at CUH.

Among patients with CPG2 prostate cancer, usage of the tool was associated with a marked increase in the rate of referral to ROPD, where the patient would be counselled on RARP as a potential treatment option and a shared treatment decision would be made. Despite a higher rate of ROPD referral when the tool was utilised, significantly fewer patients in this risk sub‐category opted for RARP, independent of the patient's age or disease characteristics. For men with CPG3 prostate cancer, the use of Predict Prostate predicted a lower likelihood of RARP after controlling for the effect of age and disease characteristics on treatment choice. Our hypothesis that tools such as Predict Prostate may encourage patients with intermediate‐risk prostate cancer to avoid or defer radical treatment was, thus, supported.

The finding that the use of Predict Prostate altered the rate of patients with intermediate‐risk prostate cancer being referred to outpatient surgical clinic or choosing RARP requires some further examination. Patients with CPG3 prostate cancer are recommended by NICE to have radical treatment, unless they decline it.^6^ As such, it is conceivable that these patients are referred to outpatient surgical clinic after discussion in the pcSMDT, irrespective of individualised risk estimated using Predict Prostate. In the clinic, however, patients might be more influenced by their individualised risk and more likely to decline active treatment. For CPG2 disease, NICE recommends offering AS and radical treatment on an equal footing, potentially creating a space for decision aids to influence management based on individualised risk assessment both among clinicians in the MDT meeting and with patients in the outpatient clinic, albeit in different directions.^6^ Further research is needed to better understand the various influences on clinician and patient decision‐making.

The results of the present study are in contrast to a recent randomised controlled trial (RCT) that showed no overall differences in reported treatment preferences or final treatment decisions.^8^ However, it must be noted that this RCT was neither sufficiently powered nor specifically designed to evaluate the impact of Predict Prostate on final treatment decisions. Our study addresses this literature gap and is the first to evaluate the tool's effect on clinical practice on a larger scale.

As the emphasis on shared decision‐making becomes more prominent in contemporary medicine, it is essential that patients feel empowered to make informed choices pertaining to their care. To achieve this, clinicians must provide adequate decision support by carefully counselling patients on their cancer‐specific mortality risk, as well as the potential benefits and risks associated with the various treatment options.^6^

The perceived authenticity of provider communication has been shown to be the most influential mediator in patient decision making, and patients are willing to accept some decrease in survival for improvement in quality of life.^9^, ^10^ This may explain why the use of Predict Prostate, which presents patients with individualised mortality data, led to a decrease in RARP and increase in uptake of active surveillance among patients with CPG2 and CPG3 disease.

A systematic review examining 13 studies of decision aids for patients with prostate cancer found that decision aids can reduce levels of stress and anxiety but was less clear about their effect on decision choice; however, fewer patients seemed to choose surgery.^11^ Our study supports this previous data on reduction in surgery with decision aid use, among CPG2 patients specifically, which may explain why previous studies looking at all groups together were less clear.

The findings of our study are pertinent due to increasing concerns surrounding overtreatment of indolent prostate cancer with low risk of disease progression.^12^, ^13^ While the annual National Prostate Cancer Audit has reported declining rates of over‐treatment for low‐risk disease since 2014, there is scant data available on rate of radical treatment in patients with intermediate‐risk disease.^14^ The recent development and adoption of the CPG risk stratification system seek to address the heterogeneity in disease mortality in previous three‐tier risk stratification systems and thus allow for delineation between treatment recommendations for CPG2 and CPG3 patients in the United Kingdom. However, due to variation in cancer‐specific mortality within the risk group and lack of evidence supporting superiority of one treatment over the other, NICE guidelines recommend CPG2 patients to be offered all three options, namely, active surveillance, radical prostatectomy and radical radiotherapy, with no preferential recommendation.^12^ Similar recommendations are made by the American Urological Association.^15^ The lack of a clear treatment pathway for these patients reiterates the need for more widespread use of a decision aid like Predict Prostate for more standardised care. In addition, by attenuating the risk of overtreatment with better risk stratification and risk communication tools, the case for prostate cancer screening may be strengthened, given its significant potential benefits.^16^, ^17^ As the Predict Prostate tool only requires clinical parameters such as PSA, clinical stage and biopsy information, it can be easily applied in healthcare settings worldwide, including areas with limited resources.

One of the key strengths of our study is the cohort size of patients, allowing for reliable assessment of the effect of the Predict Prostate tool on treatment decisions. Second, data extraction from standardised documentation from the pcSMDT meetings ensured accuracy and minimised the number of patients excluded due to insufficient clinical data available. As national guidelines from NICE recommend that all patients eligible for radical treatment should have their care coordinated by specialist MDTs, conducting our study at a regional tertiary referral centre with a pcSMDT service provided the appropriate target population that we would expect Predict Prostate to be used for. It is worth noting that the way the surgeon conveys statistical information during clinic and the level of empathy shown could affect treatment decisions. As our study was set in a large tertiary centre, different surgeons ran the clinic visits attended by the patients included in the study. We believe this to be a strength of the study as this suggests that the Predict Prostate tool is responsible for our results, rather than one surgeon's skill in conveying the results. This gives us confidence that our results can be replicated if use of the tool is expanded to new centres.

However, we recognise the limitations of our study, including its retrospective design, and single‐centre study cohort. Additionally, we assumed that the documentation of Predict Prostate data at the patient's pcSMDT review meant that it subsequently played a part in risk communication and decision‐making at the clinic appointment. Assessing whether the statistics are only verbally communicated to patients or if they are being exposed to the online Predict Prostate tool, which has visual aids, would be important to ensure that the full benefits of the tool are being reaped. Furthermore, we did not collect data on patients who opted for radical treatment in the form of radical radiotherapy as part of this study so future work should evaluate uptake of any radical treatments, including both surgery and radiotherapy. It would also be helpful to assess the effect of Predict Prostate usage on patients' decision uncertainty and decision regret in future work. Currently, Predict Prostate is indicated for men who do not have evidence of metastasis at presentation based on diagnostic data, including conventional imaging. In the future, routine use of PSMA PET/CT could increase detection of occult metastasis, and future versions of Predict Prostate may have to be recalibrated to account for this once long‐term survival data is available.^18^

## 5 CONCLUSIONS

In summary, this study shows strong potential for Predict Prostate as a decision‐making aid both for clinicians to assess patient suitability for outpatient surgical clinic counselling and for patients to make informed treatment choices. Long‐term decision regret and appropriateness of treatment choice have yet to be assessed, which would help confirm or refute the benefit of Predict Prostate in improving management of intermediate‐risk, localised prostate cancer. Future prospective studies with clinician and patient surveys can help assess the true impact of the tool in clinical decision‐making.

## AUTHOR CONTRIBUTIONS

*Conception and design*: Nimish Shah, Vincent Gnanapragasam, and Benjamin W. Lamb. *Acquisition of data*: Madhumitha Pandiaraja, Isolde Pryle, Leah West, Lucy Gardner, Olivia Shallcross, June Tay, and Benjamin W. Lamb. *Analysis and interpretation of data*: Madhumitha Pandiaraja, Isolde Pryle, Vincent Gnanapragasam, and Benjamin W. Lamb. *Drafting of the manuscript*: Madhumitha Pandiaraja and Isolde Pryle. *Critical revision of the manuscript for importantintellectual content*: Nimish Shah, Vincent Gnanapragasam, and Benjamin W. Lamb. *Statistical analysis*: Madhumitha Pandiaraja, Isolde Pryle, and Benjamin W. Lamb. *Administrative, technical, or material support*: Leah West, Lucy Gardner, Olivia Shallcross, and June Tay. *Supervision*: Nimish Shah, Vincent Gnanapragasam, and Benjamin W. Lamb.

## CONFLICT OF INTEREST STATEMENT

MP, IP, LW, LG, OS, JT, and NS have none to declare. BWL has previously received honoraria from Astra Zeneca for speaking about the use of predict prostate. VG received honoraria from Janssen speaking about risk and prognosis in prostate cancer and use of Predict Prostate and Cambridge Prognostic Groups.
